# Supplementary material for: Resistance to Resveratrol Treatment in Experimental PTSD Is Associated with Abnormalities in Hepatic Metabolism of Glucocorticoids
Source: Int J Mol Sci. 2023 May 26;24(11):9333. doi: 10.3390/ijms24119333 (PMC10253377; doi:10.3390/ijms24119333)
Supplement: Supplementary file 1 [file ijms-24-09333-s001.zip › ijms-2401520-supplementary.pdf]

International Journal of *Molecular Sciences*

Article

Resistance to Resveratrol Treatment in Experimental PTSD Is Associated with Abnormalities in Hepatic Metabolism of Glucocorticoids

Vadim E. Tseilikman [1], Julia O. Fedotova [2], Olga B. Tseilikman [1,3], Jurica Novak [4,5,*], Marina N. Karpenko [6], Victoria A. Maistrenko [6], Svetlana S. Lazuko [7], Lyudmila E. Belyeva [8], Mustapha Kamel [9], Alexey V. Buhler [9] and Elena G. Kovaleva [9,*]

1 Scientific and Educational Center 'Biomedical Technologies', School of Medical Biology, South Ural State University, 454080 Chelyabinsk, Russia; vadimed@yandex.ru (V.E.T.); diol2008@yandex.ru (O.B.T.)
2 Laboratory of Neuroendocrinology, I.P. Pavlov Institute of Physiology RAS, 6 Emb. Makarova, 199034 Saint Petersburg, Russia; julia.fedotova@mail.ru
3 Faculty of Fundamental Medicine, Chelyabinsk State University, 454001 Chelyabinsk, Russia
4 Department of Biotechnology, University of Rijeka, 51000 Rijeka, Croatia
5 Center for Artificial Intelligence and Cyber Security, University of Rijeka, 51000 Rijeka, Croatia
6 Pavlov Department of Physiology, Institute of Experimental Medicine, 197376 Saint Petersburg, Russia; mnkarpenko@mail.ru (M.N.K.); sch_viktoriya@mail.ru (V.A.M.)
7 Department of Physiology, Vitebsk State Medical University, Frunze Av. 27, 210023 Vitebsk, Belarus; lazuko71@mail.ru
8 Department of Pathophysiology, Vitebsk State Medical University, Frunze Av. 27, 210023 Vitebsk, Belarus; lyudm.belyeva2013@yandex.ru
9 Research, Educational and Innovative Center of Chemical and Pharmaceutical Technologies Chemical Technology Institute, Ural Federal University Named after the First President of Russia B. N. Yeltsin, 620002 Ekaterinburg, Russia; mustapha.mohaab@gmail.com (M.K.); zellist@mail.ru (A.V.B.)
* Correspondence: jurica.novak@biotech.uniri.hr (J.N.); e.g.,kovaleva@urfu.ru (E.G.K.)

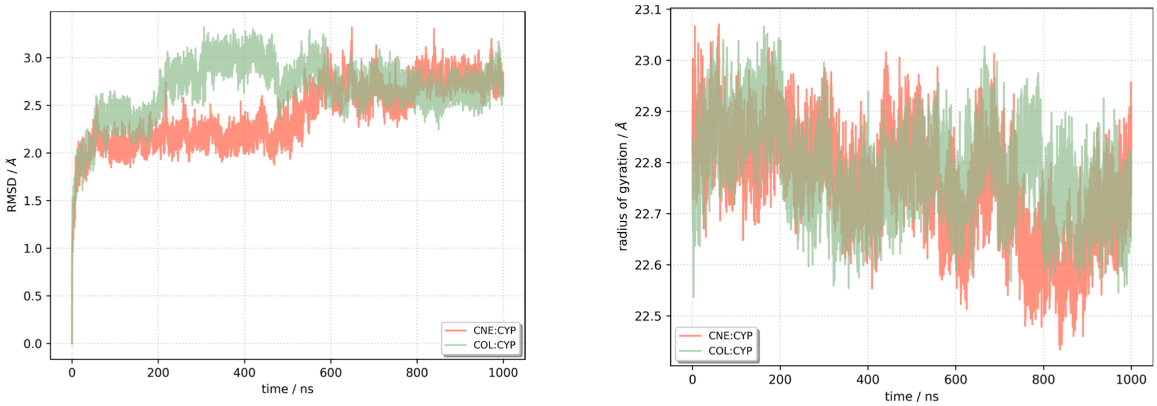

Figure S1. Stability of the COL:CYP (green) and CNE:CYP (red) complexes during molecular dynamics simulation. Root mean square deviation (left) and radius of gyration (right).

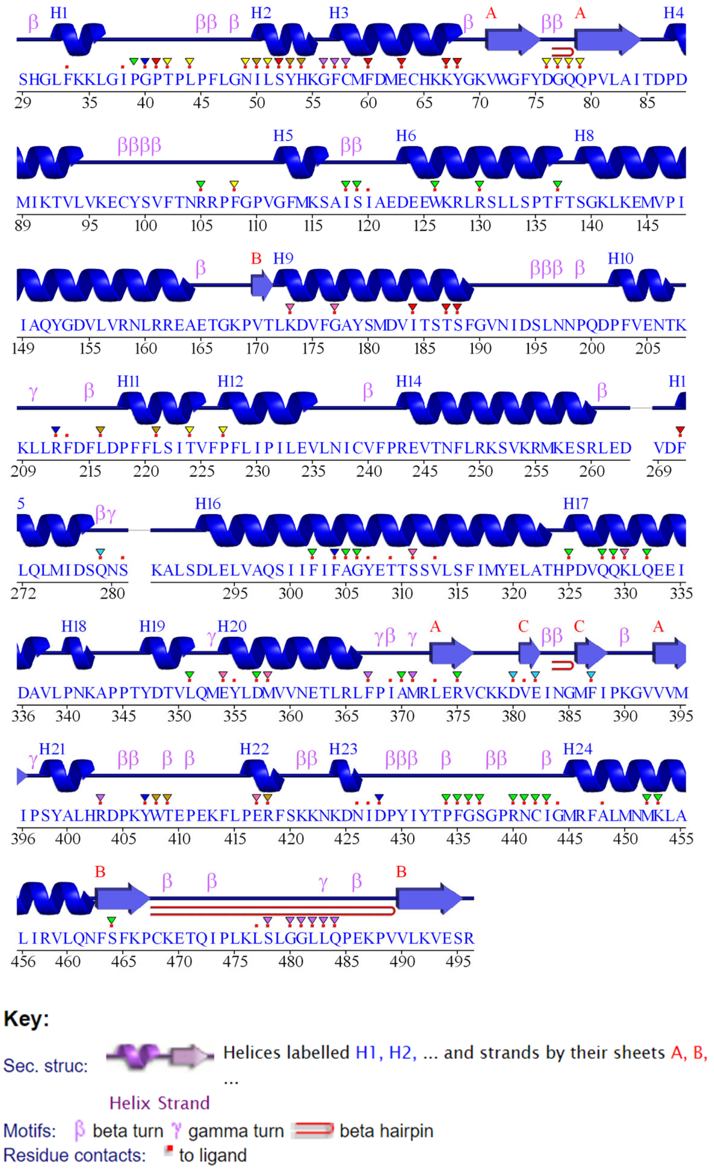

Figure S2. Secondary structure of the human CYP3A4 protein. Figure was generated with the PDBsum web server. [47.]

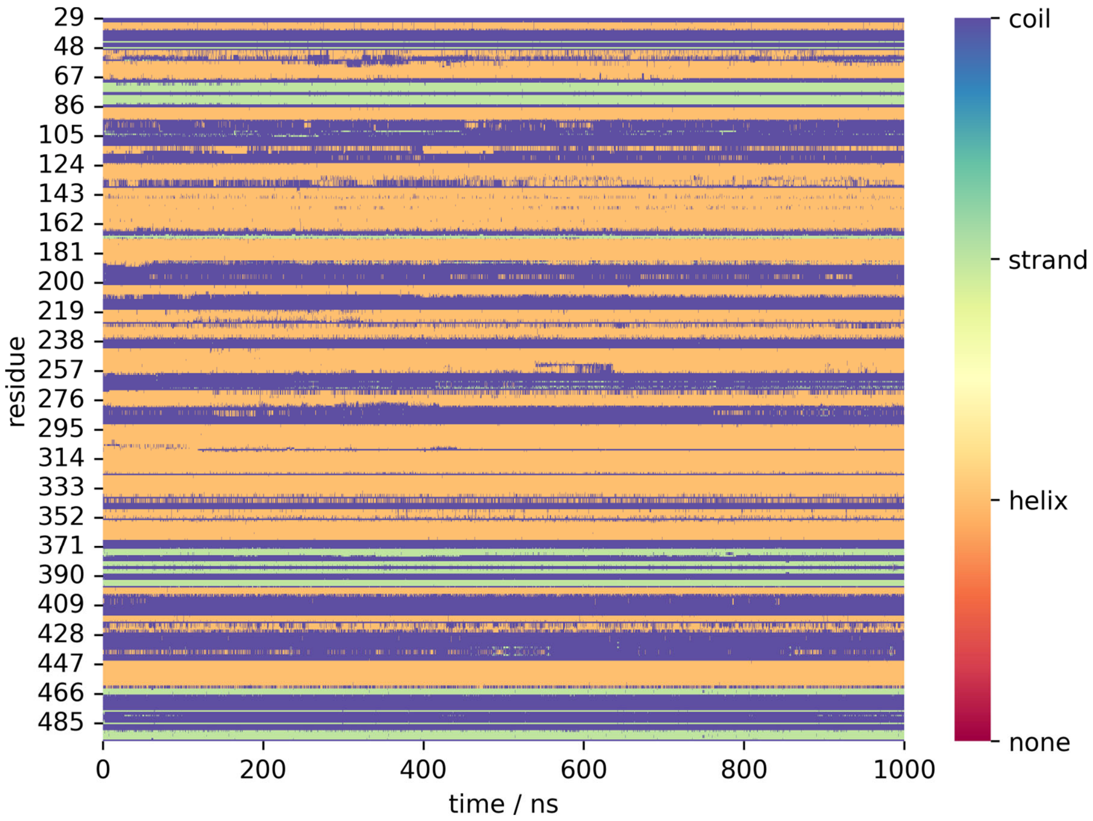

Figure S3. Changes in the secondary structure of the human CYP3A4 protein during molecular dynamics simulation.

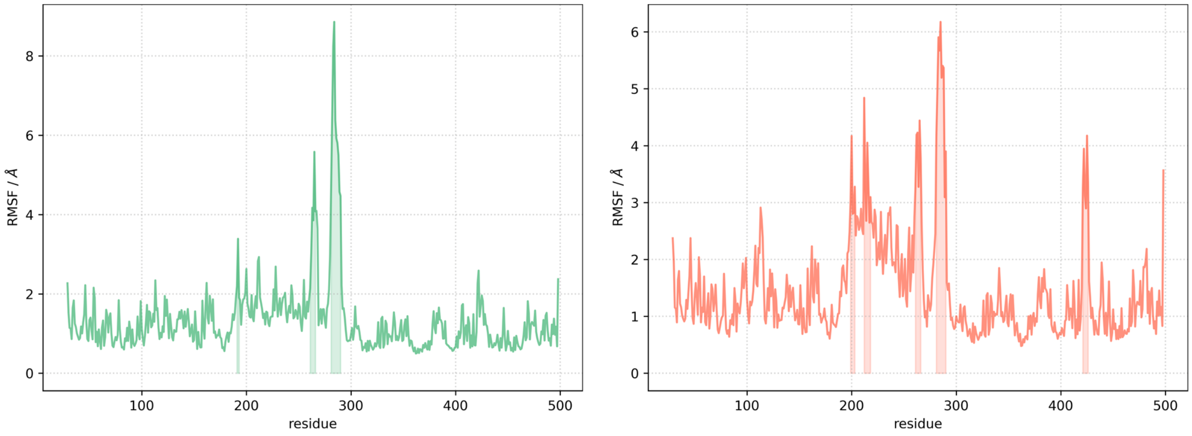

Figure S4. Root mean square fluctuations per residue of the COL:CYP (left) and CNE:CYP (right) complexes. Areas with RMSF above 3 Å are highlighted.

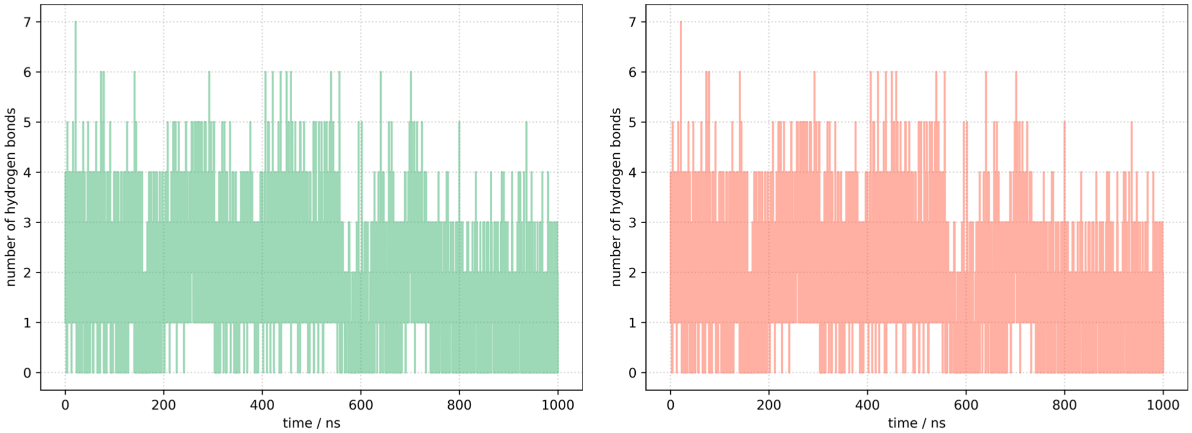

Figure S5. Number of hydrogen bond fluctuations along the COL:CYP (left) and CNE:CYP (right) trajectories.

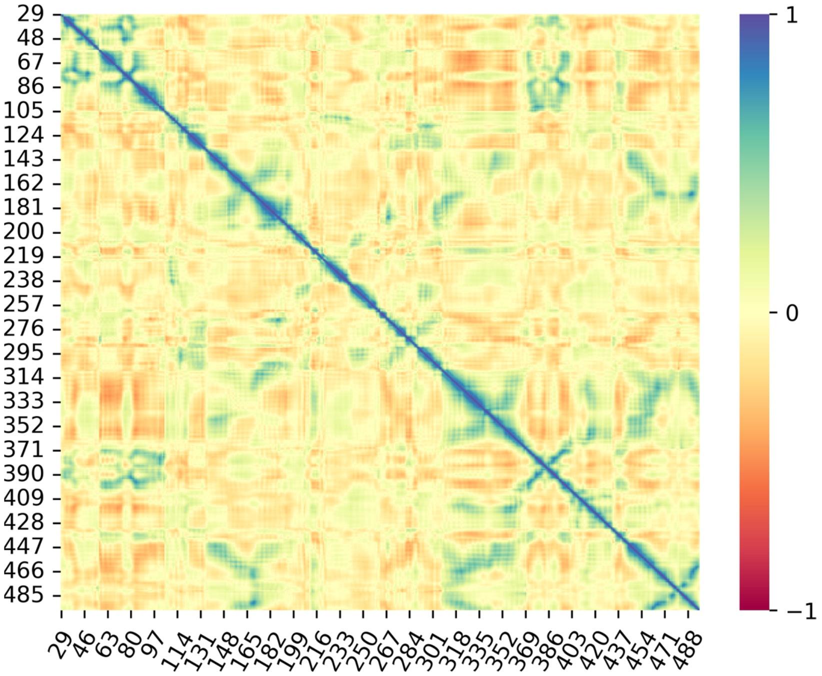

Figure S6. Dynamic cross-correlation heat map for the RES:CYP complex. Correlated movements are encoded by the red color, anti-correlated movements by the blue color.

Table S1. Clustering statistics for RES:CYP complex.

# of clusters	DBI [a]	pSF [b]	SSR/SST [c]
2	2.346	1778.5	0.151
3	2.178	1241.8	0.199
4	2.948	985.7	0.228
5	2.342	789.2	0.240
6	2.550	718.0	0.264
7	2.544	669.9	0.287
8	2.601	634.2	0.308
9	2.429	535.4	0.300
10	2.562	520.1	0.319

[a] DBI = Davies-Bouldin index, [b] pSF = pseudo-F statistic, [c] SSR/SST = ratio of sum of squares regression and sum of squares error.

Table S2. Fraction of time that the RES:CYP spends in different conformations (fraction) and the average distance to centroid (r_{AD2C}) for different numbers of clusters (k).

# cluster	fraction	r_{AD2C} / Å
	$k = 8$	
1	0.349	1.58
2	0.147	1.54
3	0.134	1.61
4	0.124	1.45
5	0.106	1.46
6	0.071	1.50
7	0.057	1.56
8	0.011	1.52
	$k = 9$	
1	0.277	1.66
2	0.256	1.50
3	0.216	1.55
4	0.110	1.46
5	0.071	1.50
6	0.051	1.52
7	0.010	1.43
8	0.007	1.36
9	0.001	1.00
	$k = 10$	
1	0.342	1.56
2	0.146	1.54
3	0.135	1.61
4	0.128	1.46
5	0.110	1.46
6	0.071	1.50
7	0.042	1.48
8	0.018	1.49
9	0.009	1.41
10	0.001	0.90

# cluster	fraction	r_{AD2C} / Å
	$k = 2$	
1	0.579	1.63
2	0.421	1.80
	$k = 3$	
1	0.579	1.63
2	0.350	1.70
3	0.071	1.68
	$k = 4$	
1	0.348	1.70
2	0.323	1.63
3	0.258	1.51
4	0.071	1.68
	$k = 5$	
1	0.466	1.59
2	0.349	1.70
3	0.113	1.47
4	0.056	1.55
5	0.016	1.60
	$k = 6$	
1	0.434	1.57
2	0.269	1.66
3	0.115	1.47
4	0.111	1.63
5	0.056	1.55
6	0.015	1.60
	$k = 7$	
1	0.432	1.57
2	0.198	1.60
3	0.117	1.64
4	0.114	1.47
5	0.071	1.50
6	0.053	1.53
7	0.015	1.59
